# Relationship between lipid parameters and vascular mechanical characteristics among a normotensive population without diabetes mellitus residing at the Qinghai–Tibet plateau: a cross-sectional study

**DOI:** 10.1186/s12872-022-02801-8

**Published:** 2022-08-05

**Authors:** Xianjin Hu, Xin Zhang, Zhipeng Zhang, Xinran Li, Qiling Gou, Runyu Ye, Xiaoping Chen

**Affiliations:** Department of Cardiology, West China Hospital, Sichuan University, Chengdu, China

**Keywords:** Pulse wave velocity, Augmentation index, Lipids, Normotensive

## Abstract

**Background:**

There is limited evidence regarding the relationship between lipid parameters and vascular mechanical characteristics in the normotensive population without diabetes mellitus. The aim of this study was to identify an association between lipid parameters and changes in vascular mechanical characteristics between men and women, and in women before and after menopause.

**Methods:**

Six hundred-seventy patients who underwent vascular functional testing and who fulfilled the inclusion and exclusion criteria were enrolled in our cross-sectional study. All participants were from the Qinghai–Tibet Plateau (Luhuo County, Ganzi Tibetan Autonomous Prefecture, Sichuan Province, China; mean altitude: 3860 m). Trained clinical physicians assessed brachial-ankle pulse wave velocity (Ba-PWV) and augmentation index adjusted to a 75-beats-per-minute heart rate (AIx@75). To investigate the relationship between lipid parameters and vascular mechanical characteristics in different sexes and menstrual stages, partial correlation analysis and multiple linear regression were used.

**Results:**

The 670 participants comprised 445 women (103 post-menopausal). Mean Ba-PWV and AIx@75 were 1315.56 ± 243.41 cm/s and 25.07% ± 15.84%, respectively. Men had greater Ba-PWV values compared with women (1341.61 ± 244.28 vs 1302.39 ± 242.17 cm/s, respectively; P < 0.05), while AIx@75 values were higher in women compared with men (27.83% ± 15.85% vs 19.64% ± 14.40%, respectively; *p* < 0.001). In the partial correlation analysis adjusted for age, total cholesterol (TC), low-density lipoprotein cholesterol (LDL-C), and non-high-density lipoprotein cholesterol (HDL-C) were associated with Ba-PWV in both men and women (*p* < 0.05); however, the magnitude was larger in men. Statistical significance was not seen for AIx@75 among both men and women. Multiple linear regression analysis revealed that TC (β = 0.165, *p* = 0.024) and non-HDL-C (β = 0.151, *p* = 0.042) remained independent predictors of change in Ba-PWV in men after adjusting for age, mean arterial pressure, waist circumference, hemoglobin, platelet count, fasting blood glucose, estimated glomerular filtration rate, and uric acid. After adjusting for traditional cardiovascular risk factors, pre-menopausal women had a similar association to that of men between LDL-C (β = 0.126, *p* = 0.030), non-HDL-C (β = 0.144, *p* = 0.013), TC/HDL-C (β = 0.162, *p* = 0.005), LDL-C/HDL-C (β = 0.142, *p* = 0.013) and Ba-PWV; however, post-menopausal women had no association between the lipid parameters and vascular function.

**Conclusions:**

Overall, TC and non-HDL-C were independent associated factors for vascular compliance alterations evaluated through Ba-PWV in normotensive men. In pre-menopausal women, LDL-C, non-HDL-C, TC/HDL-C and LDL-C/HDL-C were independent associated factors for vascular compliance alterations. After controlling for traditional risk factors, lipid profiles were not associated with these metrics for AIx@75, which can measure the amplification of reflex flow, because of the high number of confounding factors that do not genuinely reflect changes in vascular characteristics. Lipid factors did not appear to be linked to vascular function in post-menopausal women.

## Background

Hemodynamics is the study of blood flow and the vessels through which blood passes (arteries, veins, and capillaries) [1]. The intima, media, and adventitia are three concentric layers that make up the arterial wall [2]. The intima, which is the deepest layer, is made up of endothelial cells and the basement membrane, as well as other fibrous components. Smooth muscle cells and varied configurations of collagen and elastin fibers make up the intermediate layer, the media. Elastin and collagen are the major structural constituents of the adventitia, which also contains nerves and tiny arteries that supply the wall [1, 3]. The medium and large arteries are responsible for both storing blood and converting pulsatile flow into a steady flow and cushioning the damage to the peripheral microcirculation induced by pulsatile energy [4]. Hemodynamic irregularities, according to previous research [5–7], play a critical role in the onset, progression, and prognosis of cardiovascular disease, which is the major cause of early mortality and which causes enormous health and economic costs globally [8].

Pulse wave velocity (PWV) and augmentation index (AIx) are indicators that measure the velocity of forward pulsatile flow and the amplification of reflex flow, respectively, to assess the mechanical properties of vessels. Aortic PWV, carotid-femoral (Cf)-PWV, and brachial-ankle (Ba)-PWV are examples of PWV recorded at different points in the arterial tree. Because it measures the velocity of aortic blood flow, Cf-PWV is an accurate biomarker for predicting the risk of cardiovascular disease and is related to other subclinical markers [9–12]. Determining Cf-PWV has been a common approach for measuring large artery stiffness in Western countries; however, its use in studies has been limited, probably because of the limited access to this measurement method for general practitioners [13]. When compared with Cf-PWV, the most notable procedural advantage of Ba-PWV is its simplicity because it requires simply placing blood pressure cuffs on all four limbs [14]. According to previous studies, arterial stiffness of the central elastic arteries is a major determinant of Ba-PWV because there are deep links between Ba-PWV and Cf-PWV [14]. Ba-PWV measurements have been reported to have intra- and inter-observer variability of 3.8%–10.0% and 3.6%–8.4%, respectively, showing that Ba-PWV measurements are repeatable [15]. As a result, Ba-PWV is considered a reliable biomarker of large artery stiffness. AIx, which is derived from pulse wave analysis, shows how much the reflected pressure wave contributes to the ascending aortic pressure waveform [16]. AIx is calculated as the ratio of the central augmented pressure to the central pulse pressure. AIx is a complicated composite measurement impacted by age, gender, height, blood pressure, reflectance points, and left ventricular ejection parameters (such as heart rate and contractility) owing to its nature [17]. AIx@75 is AIx adjusted to a 75-beats-per-minute heart rate. Owing to the effect of reflectance points that were not associated with arterial stiffness, AIx@75 was not a surrogate marker of arterial stiffness in most studies. In this study, Ba-PWV and AIx@75 were selected as indices of the mechanical properties of large arteries; however, only Ba-PWV is a biomarker of arterial stiffness in this study.

Abnormal lipid parameters are risk factors for cardiovascular disease. The goal of this study was to investigate the link between lipid parameters and vascular mechanical characteristics in people with normal blood pressure who live on the Qinghai–Tibet Plateau.

## Methods

### Participants

This was a cross-sectional study performed by the West China Hospital that enrolled 747 normotensive individuals permanently living on the Qinghai–Tibet Plateau in Luhuo County (3860 m above sea level), Ganzi Tibetan Autonomous Prefecture, Sichuan Province, from December 2018 to September 2019. The inclusion criteria were as follows: (i) over 18 years of age; (ii) born and permanently living on the Qinghai–Tibet plateau; (iii) underwent Ba-PWV and AIx@75 measurements; (iv) mean systolic blood pressure (SBP) < 140 mmHg and diastolic blood pressure (DBP) < 90 mmHg on the evaluation date; and (v) no history of hypertension and diabetes mellitus. The exclusion criteria were as follows: (i) presence of severe liver, renal, or mental disorders (liver: alanine aminotransferase/aspartate aminotransferase > three times the upper limit; renal: estimated glomerular filtration rate (eGFR) < 30 mL/min/1.73 m^2^); (ii) established acute cardiovascular and cerebrovascular diseases; (iii) undergoing lipid modification, glucose-lowing, or anti-hypertension therapy; (iv) did not complete the relevant examinations and questionnaire; (v) ankle–brachial index < 0.9 or > 1.3; and (vi) individuals with other serious medical conditions for which the investigator considered the person inappropriate for inclusion in the study. After applying the inclusion and exclusion criteria, 670 participants were enrolled in this study (40 did not complete the questionnaire, 8 had severe liver dysfunction, 5 had severe renal dysfunction, and 24 had no blood laboratory data). Of the 670 participants, 390 underwent measurement of percutaneous oxygen saturation.

### Physical examination and laboratory measurements

All measurements were performed in the morning after at least 12 h of fasting state, with no smoking, or caffeine or alcohol consumption permitted. Before testing, all participants rested for at least 15 min in a quiet room at a temperature of 20 °C. Brachial blood pressure and heart rate were measured using an automated sphygmomanometer (HBP-1100; Omron Healthcare Co., Ltd., Kyoto, Japan) with patients in the supine position. Three measurements were taken at 2-min intervals, and the average of the last two measurements was calculated and recorded. Percutaneous oxygen saturation was assessed with a pulse oximeter (YX303; Yuwell, Jiangsu, China), and weight, height, waist circumference (Waist-c), and hip circumference were also measured. Height was measured by a scale ruler in centimeters (cm) and weight was measured with a weighing scale in kilograms (kg). Waist circumference was measured with an inelastic tape around navel with the person standing and feet separated about 25 cm. The measurement was done at the end of exhalation close to the skin of the abdomen. Hip circumference was measured with similar method of waist circumference determination around the bilateral iliac crest point at the highest point of protrusion of the buttocks. Fasting blood samples, collected into vacuum tubes by trained nurses, were centrifuged for 15 min, and the serum was transferred to tubes for freezing. Samples were stored at − 20 °C and transported to the West China Hospital on dry ice as soon as possible then tested in the hospital’s clinical laboratory. Lipid profiles and serum creatinine concentration were measured by an enzymatic method (ADVIA 2400; Siemens automatic biochemistry analyser) and eGFR was calculated through CKD-EPI equation according to sex, age and serum creatinine.

### Brachial-ankle pulse wave velocity

Ba-PWV measurements were performed by trained clinicians using the Vascular Profiler BP-203RPEIII (Omron). The subjects were examined after 5 min of rest in the supine position. Next, a blood pressure cuff was placed on each upper arm and each ankle of each patient, an electrocardiogram pad was clipped to each wrist. The device simultaneously recorded bilateral SBP, DBP, and Ba-PWV. Ba-PWV was calculated as the ratio of the traveled distance (which was automatically estimated from the patient’s height) divided by the transit time of the pulse wave between the brachial and posterior tibial arteries. The mean Ba-PWV values were used for analysis.

### Augmentation index

The parameters for central hemodynamics, namely central SBP, central DBP, central pulse pressure, central mean arterial pressure (MAP), augmentation pressure, and AIx, were determined noninvasively by applying a proprietary digital signal processing and transfer function using the cuff-based SphygmoCor System (AtCor Medical, Sydney, Australia). After a 5-min rest, a cuff was placed on the participant’s dominant upper arm. Brachial blood pressure and wave form were automatically determined and converted into the aortic pressure wave form using the generalized transfer function provided by the SphygmoCor software (AtCor Medical). AIx@75 was computed to standardize for a heart rate of 75 beats per minute.

### Sample size calculation

Calculation of the required sample size was performed by PASS software (Ver 21.0.3) according to the previous study [18] conducted by our team between September 2017 and May 2018 in Jiulong County, Ganzi Tibetan Autonomous Prefecture, Sichuan, China. R^2^ of multiple regression composed of Ba-PWV, total cholesterol and covariables was used to obtain a planned sample size of 630 participants to achieve 80% power (1-β) and an α of 0.05 (two tailed).

### Statistical analysis

We used IBM SPSS 25.0 (IBM Corp., Armonk, NY, USA) for all statistical analyses. All variables in this study were assessed using descriptive statistics prior to the analysis. Normality is assessed by P-P plots and Kolmogorov–Smirnov tests (KS). Continuous variables are expressed as mean and standard deviation for normally distributed variables and as median and interquartile range for non-normally distributed variables. Categorical variables are expressed as number and percentage. Partial correlation analysis was used for the correlation between arterial stiffness and the lipid parameters between genders, adjusted for age. We used multiple linear regression to analyze the risk factors for vascular mechanical properties in men and women. Statistical significance was defined as *p* < 0.05.

## Results

### Demographic characteristics

We included a total of 670 normotensive participants (33.58% men), with a mean age of 42.64 ± 12.41 years. All participants were born and lived permanently in Luhuo County on the Qinghai–Tibetan Plateau. The demographic characteristics of the study participants are shown in Table 1. The mean Ba-PWV and AIx@75 values were 1315.56 ± 243.41 cm/s and 25.07% ± 15.84%, respectively. Differences in the participants’ demographic characteristics between the sexes were significant except for age, heart rate, fasting blood glucose (FBG), eGFR, and central arterial blood pressure. Ba-PWV values were higher in men compared with women, while AIx@75 values were higher in women compared with men. Regarding the lipid parameters, the mean values for TC, LDL-C, and non-high-density lipoprotein cholesterol (non-HDL-C) were higher in men compared with women.

**Table 1:** Demographic characteristics in men and women (*n* = 670)

Variables	Women (n = 445)	Men (n = 225)	Total
Age (years)	42.96 ± 11.35	42.00 ± 14.28	42.64 ± 12.41
SBP (mmHg) ^##^	114.74 ± 11.64	120.41 ± 10.80	116.64 ± 11.67
DBP (mmHg) ^##^	72.81 ± 8.62	75.95 ± 8.01	73.87 ± 8.54
HR (bpm)	75.97 ± 11.49	76.24 ± 12.51	76.06 ± 11.83
SpO_2_ (%) ^#^	89.04 ± 4.07	87.97 ± 3.85	88.68 ± 4.03
Height (cm) ^##^	159.73 ± 6.34	169.99 ± 7.53	163.15 ± 8.31
Weight (kg) ^##^	62.93 ± 10.34	72.21 ± 13.34	65.99 ± 12.21
Waist-c (cm) ^##^	81.10 ± 11.63	88.64 ± 12.52	83.47 ± 12.41
Hip-c (cm) ^#^	96.61 ± 8.31	98.72 ± 9.76	97.27 ± 8.84
RBC (10^12/L) ^##^	4.83 ± 0.47	5.52 ± 0.53	5.06 ± 0.59
Hemoglobin (g/L) ^##^	139.02 ± 21.73	168.14 ± 19.37	148.74 ± 25.06
Platelet count (10^9/L) ^#^	248.20 ± 77.92	232.98 ± 66.33	243.12 ± 74.55
ALT (IU/L) ^##^	23.72 ± 15.27	36.72 ± 22.85	28.09 ± 19.17
AST (IU/L) ^##^	23.07 ± 7.69	25.64 ± 8.86	23.93 ± 8.18
FBG (mmol/L)	4.33 ± 1.38	4.55 ± 2.31	4.41 ± 1.75
eGFR (mL/min/1.73m^2^)	108.40 ± 13.66	107.19 ± 15.94	107.99 ± 14.46
Uric acid (µmol/L) ^##^	231.51 ± 66.05	353.62 ± 72.68	272.28 ± 89.35
TC (mmol/L)	4.83 ± 1.00	4.90 ± 1.09	4.85 ± 1.03
TG (mmol/L) ^#^	1.09 ± 0.70	1.29 ± 0.86	1.16 ± 0.76
HDL-C (mmol/L) ^##^	1.55 ± 0.30	1.32 ± 0.26	1.47 ± 0.31
LDL-C (mmol/L) ^##^	2.85 ± 0.83	3.17 ± 1.00	2.96 ± 0.90
Non-HDL-C (mmol/L) ^##^	3.28 ± 0.94	3.58 ± 1.08	3.38 ± 1.00
Ba-PWV (cm/s) ^#^	1302.39 ± 242.17	1341.61 ± 244.28	1315.56 ± 243.41
Central SBP (mmHg)	108.97 ± 11.34	109.72 ± 9.99	109.22 ± 10.90
Central DBP (mmHg)	76.18 ± 9.03	77.48 ± 8.68	76.62 ± 8.93
MAP (mmHg)	90.04 ± 11.16	88.21 ± 16.03	89.43 ± 13.02
PP (mmHg)	33.41 ± 10.33	35.17 ± 15.09	34.00 ± 12.16
AP (mmHg) ^##^	9.80 ± 7.21	6.83 ± 5.47	8.80 ± 6.82
AIx@75 (%)^##^	27.83 ± 15.85	19.64 ± 14.40	25.07 ± 15.84

^#^*p* < 0.05; ^##^*p* < 0.001

*SBP* systolic blood pressure, *DBP* diastolic blood pressure, *SpO*_2_ percutaneous oxygen saturation, *Waist-c* Waist circumference, *Hip-c* Hip circumference, *RBC* Red blood cell count, *ALT* Alanine aminotransferase, *AST* Aspartate aminotransferase, *FBG* Fasting blood glucose, *eGFR* Estimated glomerular filtration rate, *TC* Total cholesterol, *TG* Triglycerides, *HDL-C* High-density lipoprotein cholesterol, *LDL-C* Low-density lipoprotein cholesterol, *Ba-PWV* Brachial-ankle pulse wave velocity, *MAP* Mean arterial pressure, *PP* Pulse pressure, *AP* Augmentation pressure, *AIx* Augmentation index

### Partial correlation analysis of the relationship between vascular characteristics and the lipid parameters

The relationships between vascular function and the lipid parameters between women and men are listed in Tables 2 and 3, respectively. In women, Ba-PWV showed a significant positive correlation with TC (r = 0.105, *p* = 0.026), LDL-C (r = 0.109, *p* = 0.021), non-HDL-C (r = 0.120, *p* = 0.011), TC/HDL-C (r = 0.104, *p* = 0.028), adjusted for age. In comparison, in men, Ba-PWV was positively correlated with TC (r = 0.161, *p* = 0.016), LDL-C (r = 0.135, *p* = 0.044), and non-HDL-C (r = 0.148, *p* = 0.027), adjusted for age. Notably, AIx@75 was not correlated with the lipid parameters and their ratios after adjusting for age, regardless of sex.

**Table 2:** Partial correlation analysis of the relationship between vascular characteristics and lipids in women

Variables	Ba-PWV		AIx@75	
	r	*p*	r	*p*
TC	0.105	0.026^#^	− 0.050	0.295
TG	0.092	0.053	0.055	0.248
HDL-C	− 0.026	0.585	− 0.010	0.833
LDL-C	0.109	0.021^#^	− 0.056	0.240
Non-HDL-C	0.120	0.011^#^	− 0.050	0.300
TC/HDL-C	0.104	0.028^#^	− 0.006	0.893
TG/HDL-C	0.088	0.063	0.078	0.104
LDL-C/HDL-C	0.092	0.054	− 0.017	0.722

^#^*p* < 0.05

Partial correlation coefficients were calculated after adjusting for age

*TC* Total cholesterol, *TG* Triglycerides, *HDL-C* High-density lipoprotein cholesterol, *LDL-C* Low-density lipoprotein cholesterol

**Table 3:** Partial correlation analysis of the relationship between vascular characteristics and lipids in men

Variables	Ba-PWV		AIx@75	
	r	*p*	r	*p*
TC	0.161	0.016^#^	0.076	0.262
TG	0.081	0.227	0.019	0.777
HDL-C	0.041	0.539	− 0.024	0.722
LDL-C	0.135	0.044^#^	0.069	0.311
Non-HDL-C	0.148	0.027^#^	0.081	0.232
TC/HDL-C	0.079	0.240	0.100	0.139
TG/HDL-C	0.050	0.461	0.034	0.612
LDL-C/HDL-C	0.077	0.253	0.096	0.154

^#^*p* < 0.05

Partial correlation coefficients were calculated after adjusting for age

*TC* Total cholesterol, *TG* Triglycerides, *HDL-C* High-density lipoprotein cholesterol, *LDL-C* Low-density lipoprotein cholesterol

### Multiple linear regression analysis of the relationship between Ba-PWV and the lipid parameters

In the multiple linear regression analysis, we used different models, including conventional cardiovascular risk factors and one of lipid parameters. Each linear regression model was adjusted for age, MAP, Waist-c, hemoglobin, platelet count, FBG, eGFR, and uric acid. As shown in Table 4, TC (β = 0.165, *p* = 0.024) and non-HDL-C (β = 0.151, *p* = 0.042) were independent risk factors associated with Ba-PWV in men. However, in women, the lipid parameters evaluated in this study were not statistically significantly associated with Ba-PWV. In a subset analysis, we divided the women into a pre-menopausal group and a post-menopausal group. The demographic characteristics of these two groups are shown in Table 5. We also performed multiple linear regression analysis of the relationship between Ba-PWV and the lipid parameters in these two groups (Table 6). In the pre-menopausal group, LDL-C (β = 0.126, *p* = 0.030), non-HDL-C (β = 0.144, *p* = 0.013), TC/HDL-C (β = 0.162, *p* = 0.005) and LDL-C/HDL-C (β = 0.142, *p* = 0.013) were risk factors associated with Ba-PWV after adjusting for age, MAP, Waist-c, hemoglobin, platelet count, FBG, eGFR, and uric acid. In the post-menopausal group, the lipid parameters included in this study had no statistically significant association with Ba-PWV.

**Table 4:** Multiple linear regression analysis of the relationship between Ba-PWV and lipids

Variables	B	β (sta)	*p*
*Women*			
TC	10.837	0.046	0.327
TG	25.246	0.077	0.084
HDL-C	− 40.276	− 0.052	0.224
LDL-C	13.169	0.046	0.328
Non-HDL-C	17.501	0.071	0.138
TC/HDL-C	25.671	0.083	0.073
TG/HDL-C	39.064	0.082	0.070
LDL-C/HDL-C	21.482	0.062	0.176
*Men*			
TC^#^	35.672	0.165	0.024
TG	11.315	0.044	0.541
HDL-C	62.265	0.071	0.302
LDL-C	18.317	0.080	0.301
Non-HDL-C^#^	33.140	0.151	0.042
TC/HDL-C	12.176	0.052	0.483
TG/HDL-C	0.952	0.004	0.961
LDL-C/HDL-C	1.464	0.006	0.940

^#^ *p* < 0.05

Adjusted for age, MAP, Waist-c, hemoglobin, platelet count, FBG, eGFR, and uric acid

*TC* Total cholesterol, *TG* Triglycerides, *HDL-C* High-density lipoprotein cholesterol, *LDL-C* Low-density lipoprotein cholesterol, *MAP* Mean arterial pressure, *Waist-c* Waist circumference, *FBG* Fasting blood glucose, *eGFR* Estimated glomerular filtration rate

**Table 5:** Demographic characteristics of the pre- and post-menopausal groups

Variables	Pre-menopausal (n = 342)	Post-menopausal (n = 103)
Age^##^	38.88 ± 8.57	56.50 ± 8.63
MAP	89.53 ± 11.71	91.75 ± 8.91
Waist-c^##^	79.88 ± 11.19	85.82 ± 11.88
Hemoglobin^##^	136.66 ± 22.79	147.06 ± 15.20
Platelet count^#^	253.76 ± 83.26	229.19 ± 52.00
FBG	4.27 ± 1.36	4.52 ± 1.45
eGFR^##^	111.95 ± 12.28	96.60 ± 11.17
Uric acid^#^	226.87 ± 63.57	246.94 ± 71.89
TC^##^	4.67 ± 0.96	5.35 ± 0.96
TG^#^	1.03 ± 0.67	1.29 ± 0.75
HDL-C	1.54 ± 0.30	1.60 ± 0.30
LDL-C^#^	2.73 ± 0.78	3.22 ± 0.88
Non-HDL-C^##^	3.13 ± 0.89	3.75 ± 0.95
Ba-PWV^##^	1248.97 ± 179.90	1479.76 ± 325.20
AIx@75^##^	25.24 ± 14.90	36.56 ± 15.88

^#^*p* < 0.05, ^##^*p* < 0.001

*MAP* Mean arterial pressure, *Waist-c* Waist circumference, *FBG* Fasting blood glucose, *eGFR* Estimated glomerular filtration rate, *TC* Total cholesterol, *TG* Triglycerides, *HDL-C* High-density lipoprotein cholesterol, *LDL-C* Low-density lipoprotein cholesterol, *Ba-PWV* Brachial-ankle pulse wave velocity, *AIx@75* Augmentation index adjusted to a 75-beats-per-minute heart rate

**Table 6:** Multiple linear regression of Ba-PWV and lipids in the pre- and post-menopausal groups

Variables	B	β (sta)	*p*
*Pre-menopausal*			
TC	19.602	0.105	0.068
TG	21.417	0.082	0.143
HDL-C	− 40.530	− 0.069	0.203
LDL-C^#^	29.313	0.126	0.030
Non-HDL-C^#^	28.992	0.144	0.013
TC/HDL-C^#^	41.563	0.162	0.005
TG/HDL-C	38.572	0.099	0.082
LDL-C/HDL-C^#^	40.259	0.142	0.013
*Post-menopausal*			
TC	− 16.216	− 0.049	0.564
TG	26.419	0.066	0.439
HDL-C	29.800	0.028	0.732
LDL-C	− 28.207	− 0.080	0.368
Non-HDL-C	− 19.503	− 0.059	0.490
TC/HDL-C	− 23.703	− 0.065	0.448
TG/HDL-C	22.569	0.041	0.639
LDL-C/HDL-C	− 32.214	− 0.081	0.359

^#^*p* < 0.05

Adjusted for age, MAP, Waist-c, hemoglobin, platelet count, FBG, eGFR, and uric acid

*TC* Total cholesterol, *TG* Triglycerides, *HDL-C* High-density lipoprotein cholesterol, *LDL-C* Low-density lipoprotein cholesterol, *Ba-PWV* Brachial-ankle pulse wave velocity, *MAP* Mean arterial pressure, *FBG* Fasting blood glucose, *eGFR* Estimated glomerular filtration rate

## Discussion

The purpose of this study was to evaluate the association between lipid parameters and the mechanical properties of the walls of large arteries in normotensive individuals without diabetes mellitus living on the Qinghai–Tibet Plateau. In our study, all participants lived at high altitude area and hypobaric hypoxia exposure and highly saline diets produced variety effect in vasculature system which is distinctive compared with population living at sea level area. Indeed, there were many confounding factors such as blood pressure and glucose. Hence, we enrolled participants without hypertension and diabetes mellitus to avoid a major part of confounding factors. We confirmed that the relationship between lipid parameters and vessels, particularly regarding Ba-PWV, was associated with gender in this cross-sectional investigation. Specifically, after adjusting for age, MAP, Waist-c, hemoglobin, platelet count, FBG, eGFR, and uric acid, Ba-PWV was positively associated with TC and non-HDL-C in men. In contrast, women did not have these associations. Only pre-menopausal women showed a comparable positive relationship between LDL-C, non-HDL-C, and Ba-PWV in the subgroup analysis of pre-menopausal women and post-menopausal women. Additionally, our investigation identified no such associations for AIx@75 in either men or women. Previous investigations of the association between lipids and vessel mechanical characteristics came to different conclusions. After controlling for age, gender, and ethnicity, Havlik et al. observed that Cf-PWV was negatively associated with HDL-C (p = 0.003) but not with TC, triglycerides, and LDL-C [19]. Cf-PWV was associated with LDL-C when adjusted for age, gender, MAP, heart rate, and Waist-c in Zhao et al.'s investigation in a Chinese population [20]. The results of a correlation analysis by Yadav et al. [20] showed that Cf-PWV was moderately associated with triglycerides, HDL-C, and the ratio of these two lipid parameters in a young Korean population [21]. The differences in the results of these studies might be explained by differences in sample characteristics (such as race, sex ratio, and age). Among participants in our research, TC and non-HDL-C seemed to be better biomarker for increment of arterial stiffness in men compared with conventional lipid parameters and their ratios. Furthermore, complications may change vascular characteristics, based on particular lipid parameters. Additionally, the concentration of LDL-C and apolipoprotein B in type 1 diabetes mellitus patients may play a key role in endothelial functional impairment [22, 23]. The concentration of apolipoprotein B was a main determinant of elevated Cf-PWV in participants with phase I–III chronic renal disease in one study [24]. The combination of hypertension and hyperlipidemia leads to subtle changes in the morphological and functional characteristics of the arterial wall, as measured by PWV and brachial Aix [25]. The dysfunctional arterial wall, including endothelial and tunica media function, improved with appropriate lipid-lowering medication in several studies [26–29]. LDL-C appeared to be the fundamental cause of vessel wall degeneration, and TG was the next most common reason for dysfunction when LDL-C was at the target level. In pre-menopausal population recruited in our study, we found that LDL-c and non-HDL-C were prominent lipid parameters which associated with vascular characteristics. Nevertheless, in type 2 diabetes mellitus, the amelioration of flow-mediated vasodilatation through combined treatment with fenofibrate and ezetimibe was significantly associated with elevated HDL-C subfractions [30]. A cohort study of type 2 diabetes mellitus patients with LDL-C ≤ 2.0 mmol/L confirmed a relationship between HDL-C and vascular events and all-cause death [31]. To prevent the effect of these confounding variables, we centered our study on a normotensive group without type 2 diabetes mellitus. We found that lipid parameters were associated with the mechanical properties of the walls of large arteries, as assessed by Ba-PWV, which is a widely accepted indicator of arterial stiffness. Similarly, previous studies confirmed that LDL-C, HDL-C, and TC were associated with PWV in the normotensive population [32, 33]. According to studies by Koschinsky et al. and Sanyour et al. [34, 35], lipids could stimulate the detachment and migration of vascular smooth muscle cells, which is a crucial step in altering the properties of vessels.

Sex was one of the most important factors affecting the properties of the vascular wall. In our study, we observed that TC, LDL-C, and non-HDL-C were positively correlated with Ba-PWV among men in the partial correlation analysis adjusted for age. However, in the multiple linear regression models, only TC and non-HDL-C were risk factors for stiffness in large arteries after adjusting for conventional cardiovascular risk factors. In comparison, in women, TC, LDL-C, non-HDL-C, and TC/HDL-C were positively corelated with Ba-PWV in the partial correlation analysis. After adjusting for conventional risk factors, the relationship between lipids and arterial stiffness was not statistically significant among women. Previous studies also noticed a sex difference in the circulatory system and suggested that sex hormones were a crucial factor related to this phenomenon. Some clinical trials also assessed the influence of estrogen replacement therapy on vascular properties in post-menopausal women. A few randomized controlled trials and crossover studies investigated the effect of hormone replacement therapy on lipid profiles and vascular function. The studies found that transdermal and oral hormone replacement therapy improved dyslipidemia but not vascular wall characteristics during 3 months to 2 years of follow-up [36–39]. However, recently, two small sample-size randomized controlled trials showed that the combination of estradiol and dydrogesterone/drospirenone reduced PWV among post-menopausal women [40, 41]. In our study, we found that LDL-C and non-HDL-C were positively associated with stiffness in large arteries in pre-menopausal women after adjusting for conventional cardiovascular risk factors. However, in post-menopausal women, Ba-PWV and AIx@75 were not associated with the lipid parameters after adjusting for conventional risk factors. This finding suggests that lipid parameters might not be one of the most vital risk factors in vascular dysfunction in post-menopausal women. According to previous studies, it was unclear whether estrogen had a prominent influence on vascular functional alterations except regarding conventional risk factors, in post-menopausal women. Hence, lipid lowing therapy might not improve vascular mechanical properties in large vessels in normotensive post-menopausal women without type 2 diabetes mellitus.

Several limitations of this study should be mentioned. First, this was a cross-sectional study, and the results cannot demonstrate causality. Hence, our findings should be verified in future cohort studies. Second, we did not evaluate certain other conventional lipid parameters, such as apolipoprotein B. Finally, some participants in our study had impaired glucose tolerance, which cannot be estimated by FBG, and impaired glucose tolerance may affect vessel function.

## Conclusions

The effect of lipid parameters on the properties of large vessels differed in normotensive men and women. TC and non-HDL-C were independent associated factors for vascular compliance alterations evaluated through Ba-PWV in normotensive men. In pre-menopausal women, LDL-C, non-HDL-C, TC/HDL-C and LDL-C/HDL-C were independent associated factors for vascular compliance alterations. In post-menopausal women, lipid parameters appear to be unrelated to vascular function. Hormonal changes might partly account for this phenomenon. Regarding AIx@75, which can assess the amplification of reflex flow, lipid parameters were not related to this index after adjusting for conventional risk factors because of the high number of confounding factors that do not reflect alterations in vascular mechanical properties.

## Data Availability

The datasets used in the present study are available from the corresponding author on reasonable request.

## Abbreviations

AIx: Augmentation index
AIx@75: AIx normalized for a heart rate of 75 beats per minute
ALT: Alanine aminotransferase
AP: Augmentation pressure
AST: Aspartate aminotransferase
Ba-PWV: Brachial-ankle pulse wave velocity
Cf-PWV: Carotid-femoral pulse wave velocity
DBP: Diastolic blood pressure
eGFR: Estimated glomerular filtration rate
FBG: Fasting blood glucose
HDL-C: High-density lipoprotein cholesterol
LDL-C: Low-density lipoprotein cholesterol
MAP: Mean arterial pressure
PP: Pulse pressure
RBC: Red blood cell count
SBP: Systolic blood pressure
TC: Total cholesterol
TG: Triglycerides
Waist-c: Waist circumference
